# Distribution of ganciclovir in the porcine central nervous system

**DOI:** 10.1128/aac.01815-24

**Published:** 2025-03-21

**Authors:** Johan Mikkel Guldbæk, Theis Mariager, Mikkel Dreyer Nielsen, Jacob Holmen Terkelsen, Roland Nau, Carsten Reidies Bjarkam, Henrik Nielsen, Jacob Bodilsen

**Affiliations:** 1 Department of Clinical Medicine, Aalborg University 572587, Aalborg, North Denmark, Denmark; 2 Department of Infectious Diseases, Aalborg University Hospital 669295, Aalborg, North Denmark, Denmark; 3 ESCMID Study Group for Infectious Diseases of the Brain (ESGIB), Basel, Switzerland; 4 Department of Neurosurgery, Aalborg University Hospital 53141, Aalborg, North Denmark, Denmark; 5 Institute of Neuropathology, University Medical Centre 27177, Göttingen, Lower Saxony, Germany; IrsiCaixa Institut de Recerca de la Sida, Barcelona, Spain

**Keywords:** microdialysis, pharmacokinetics, ganciclovir, animal models, antiviral agents, antiviral pharmacology, CNS

## Abstract

Ganciclovir is often used compassionately for encephalitis due to cytomegalovirus (CMV) and human herpes virus 6b (HHV-6b). Ganciclovir pharmacokinetic studies in the central nervous system (CNS) generally rely on single measurements in the cerebrospinal fluid (CSF) or homogenized brain tissue. Therefore the objective was to compare brain extracellular fluid (ECF) concentrations of ganciclovir with plasma and CSF concentrations in a porcine model, using microdialysis during a 24 h period. Six Danish landrace pigs (female, age 4 months, 31–37 kg) received two weight-adjusted intravenous doses of ganciclovir. Unbound ganciclovir concentrations were determined by microdialysis over 24 h in five compartments: CSF (lateral ventricle, cisterna magna, and lumbar) and brain ECF (cortical and subcortical). Data were compared with paired plasma samples. Ganciclovir concentrations >IC_50_ for CMV (1.6 µg/mL) were achieved in all compartments. Concentrations >IC_90_ for CMV (8.3 µg/mL) were only achieved in plasma and the lumbar CSF compartment. The concentration time curves indicated higher lumbar and cisternal CSF concentrations than ECF concentrations. The ECF compartments achieved greater maximum concentration (C_max_), area under the concentration time curve (AUC), and time >IC_50_ after the second dose, and an accumulation ratio (R_ac_) >1. The greater C_max_, AUC, time >IC_50_, and R_ac_ >1 in the ECF compartments with repeated dosages suggest that therapeutic concentrations may be achieved during long-term treatment. A higher loading dose might be warranted to improve early viral inhibition.

## INTRODUCTION

Ganciclovir is a viral DNA polymerase inhibitor used for treatment of encephalitis and other severe infections due to cytomegalovirus (CMV) and human Herpesvirus 6b (HHV-6b). Risk groups include neonates, solid organ transplant recipients, patients with AIDS, and hematological malignancies (1–9). However, the clinical benefit of ganciclovir in patients with CMV or HHV-6b encephalitis remains controversial, and debilitating neurological sequelae often persist among the survivors (1, 8, 10–14). Furthermore, ganciclovir has a high interpatient pharmacokinetic (PK) variability and is associated with considerable risks of bone marrow and renal toxicity (1, 15–17).

Current guidelines for treatment of CMV infections suggest intravenous (IV) ganciclovir at an initial dose of 5 mg/kg every 12 h for 14–21 days. In HIV patients, this should be followed by a maintenance dose of 5 mg/kg every 24 h until the CD4+ count is restored to >100 cells/mm^3^ for at least 3–6 months (9, 11, 18, 19).

The antiviral susceptibility of CMV to ganciclovir can be determined through different methods including *in vitro* plaque reduction assays. From this, an inhibitory concentration is expressed, most often the 50% inhibitory concentration (IC_50_) or 90% inhibitory concentration (IC_90_) (20). Studies have reported varying CMV IC_50_s of ganciclovir from 0.4 to 1.6 µg/mL in drug sensitive isolates, to well above 2 µg/mL in resistant isolates (20–27). The IC_90_ has been reported to range from 0.5 to 16.5 µg/mL (27–30). Unfortunately, the IC_50_ of human hematopoietic progenitor cells is relatively low (0.69 µg/mL) (31), which provides a high risk of myelosuppression during antiviral therapy.

Previous PK studies of ganciclovir for central nervous system (CNS) infections usually relied on single cerebrospinal fluid (CSF) measurements (32–35) or homogenized brain tissue sampling (36, 37). The reported human lumbar CSF concentrations are 0.5–8.5 µg/mL for the maximum concentration (C_max_) (34, 35), 2.6 µg/mL × h for the area under the curve (AUC) (35) and 0.24–0.67 for the penetration ratio (34). Yet, virions reside and replicate within neurons, and homogenized tissue samples may contain residual blood which may substantially obscure drug tissue concentrations. Therefore, continuous sampling of brain extracellular fluid (ECF) may be more representative of the intracellular drug concentrations.

This study aimed to determine and compare brain ECF concentrations of ganciclovir with plasma and CSF concentrations in a porcine model, using microdialysis during a 24 h period.

## MATERIALS AND METHODS

The study was conducted at Aalborg University Hospital, Denmark. Ethical approval was obtained by the Danish Animal Experiments Inspectorate and carried out in accordance with existing laws (License no. 2020-15-0201-00401).

### Overview

The intervention and sampling procedure have been tested and validated in a previous PK study (38). The current study was performed on six Danish landrace pigs (female, age 4 months, 31–37 kg). All animals received a total of two weight-adjusted intravenous boluses of ganciclovir (Cymevene; Cheplapharm, Greifswald, Germany) of 5 mg/kg. Doses were administered at 0 h and 12 h, respectively, to best emulate current dosage recommendations (5 mg/kg every 12 h). Drug concentrations were determined through microdialysis over 24 h in five compartments: CSF (lateral ventricle, cisterna magna, and lumbar) and brain ECF (cortical and subcortical). For reference, plasma drug concentrations were also measured at each sampling from the CNS.

### Microdialysis

Microdialysis is a method that allows continuous sampling of different molecules in live tissues or fluid-filled spaces (39). A probe equipped with a semipermeable membrane is inserted into a desired tissue. The semipermeable membrane allows passive diffusion of molecules with a certain molecular weight (low-high), which enables certain target molecules to be selectively measured with minimal disturbance of the surrounding tissue (40). A perfusate of 0.9% NaCl (Fresenius Kabi AB, Uppsala, Sweden) is delivered through the probe at a constant flow rate of 1 µL/min, which prevents equilibrium across the membrane. The dialysate, consisting of the perfusate containing the desired compound after being in contact with the semipermeable membrane, is then collected.

The dialysate will only contain a fraction of the true extracellular concentration of the target molecule. To calculate the actual tissue concentration, the retrodialysis-by-drug approach (41, 42) was utilized. The relative recovery (RR) was determined *in vivo* by individual catheter-specific calibration and then calculated through the following equation:


$$RR\left(\%\right)=100\times \left(1-\frac{C_{dialysate}}{C_{perfusate}}\right).$$

C_dialysate_ is the concentration of ganciclovir (mg/L) in the dialysate, and C_perfusate_ is the concentration of ganciclovir (mg/L) in the perfusate. During data analysis, the measured concentrations were attributed to the midpoint of the sampling intervals.

The total tissue concentration (C_tissue_) was then determined by correcting for the membrane-specific RR of the drug through the following equation:


$$C_{tissue}=100\times \frac{C_{dialysate}}{RR\left(\%\right)}.$$

#### Intervention

##### Anesthesia and monitoring

The animals were anesthetized by intramuscular administration of 6 mL midazolam (5 mg/mL) and 4 mL ketamine (25 mg/mL), intubated (Portex tube 6-5 Smiths Medical, UK) and mechanically ventilated. A 6 FR catheter sheath (Avanti, Cordis Cashel, Ireland) was inserted into the right jugular vein for venous blood sampling and infusion of ganciclovir, fentanyl (50 µg/mL), propofol (10 mg/mL), and NaCl (0.9%). A separate catheter was inserted in the right femoral artery for arterial blood sampling and continuous pulse and blood pressure monitoring. A thermal sensor catheter was inserted into the bladder for continuous temperature measurements. The porcine homeostatic temperature range of 36.5°C–39°C was maintained throughout the entire procedure, using a forced-air warming blanket. Blood pH was kept between 7.45 and 7.59.

##### Surgical procedure

The surgical procedure and insertion of microdialysis catheters have previously been described in detail (38, 43). Briefly, an anterior craniectomy allowed the insertion of separate microdialysis catheters in three compartments: the cerebral cortex, subcortical white matter, and lateral ventricle. Two microdialysis catheters were inserted in the cisterna magna and the lumbar spinal canal (L4/L5) through two posterior incisions. Correct placement of all CSF catheters was verified by aspiration of CSF prior to microdialysis catheter insertion.

### Data collection

Prior to drug administration, tissue equilibrium was achieved during a 40 min waiting period (44). Then a weight-adjusted IV dose of ganciclovir (5 mg/kg) was administered at 0 h (155–185 mg). Samples were collected in 30 min intervals from 0 to 2 h, in 60 min intervals from 3 to 4 h, and 120 min intervals from 4 to 12 h. At 12 h a second identical intravenous dosage was given, and the samplings were collected in 30 min intervals from 12 to 13 h and in 120 min intervals from 14 to 24 h. Venous blood samples were drawn at the midpoint of each interval. The aim was to draw 105 microdialysis samples and 18 plasma samples from each animal (738 total samples), but only 697 samples were eligible for analysis (589 microdialysis and 108 plasma samples). An overview of the sampling intervals can be found in the supplementary materials. After collection of all samples, each microdialysis catheter was calibrated through *in vivo* retrodialysis performed as triplets at 100 µg/mL. Venous blood samples were stored at 5°C for 10 min before being centrifuged for 15 min at 7°C/1,500 g. Dialysates and plasma aliquots were kept on dry ice during the procedure and thereafter stored at −80°C until analysis.

### Quantification of ganciclovir concentrations

Dialysate and plasma concentrations of unbound ganciclovir were analysed by high-pressure liquid chromatography mass spectrometry as described earlier (38) with a lower limit quantification of (0.01 µg/mL). Validation of the analytical model was performed in regard to sensitivity, linearity, accuracy, and precision with correlation coefficients (R^2^) >0.99 and coefficients of variation <5% for all QC levels. Chemical analyses were performed at the facilities of BioXpedia A/S, Aarhus, Denmark (see supplementary material).

### Antiviral target definition

The IC_50_ and IC_90_ values presented in this study were derived from the current literature (20–30). The upper end of the sensitive IC_50_ range (1.6 µg/mL) was utilized in this study. Due to the wide range of IC_90_ values, this study utilized the mid-range value of 8.3 µg/mL. Since there is no standardized PK/PD model for ganciclovir treatment of CMV, the AUC and time above IC_50_ and IC_90_ were evaluated instead. Since most models define antiviral activity as concentration >IC_50_ (21–27), this was chosen as the primary target, with the IC_90_ as a secondary analysis. Both the t > IC_50_ and t > IC_90_ were determined through interpolation between data points.

### Data analysis

As the plasma protein binding of ganciclovir is deemed negligible (1%–2%) (15), unbound concentrations of ganciclovir were determined in plasma and all CNS compartments. Categorical variables were summarized as n/N. Ganciclovir concentrations were presented as medians and ranges, due to the small sample size of animals (*n* = 6). A significance level of 5% (*P* ≥ 0.05) was utilized.

Non-compartmental analysis was performed for each compartment in each animal. The following PK parameters were calculated for the first and second dose, respectively: maximum concentration (C_max_), AUC, half-life (T_1/2_), and tissue penetration ratios. The AUCs were determined from drug administration to the last measurement (AUC_0–12 h_ and AUC_12–24 h_) for each dose. The AUCs were calculated using the linear trapezoidal rule (45). The tissue penetration ratio was determined by comparing the AUC of each CNS compartment to the plasma AUC (AUC_tissue_/AUC_plasma_). Complete tissue penetration was defined as a penetration ratio >0.8. The accumulation ratio (R_ac_) was used as an expression for drug accumulation in each CNS compartment and defined as the ratio between the second and first dose. Elimination rate constants (k) were estimated by log-linear regression, and elimination half-lives (T_1/2_) were calculated as ln 2/k.

Due to a non-normal distribution and a small sample size, the median concentrations and PK parameters were compared using non-parametric pairwise comparisons via Wilcoxon signed rank test, with Bonferroni correction. The model assumptions were tested by visual examination of residuals, fitted values, and estimates of random effects. Missing data points were handled through interpolation. The PK parameters and statistical analyses were performed in Stata (v. 18, StataCorp LLC, College Station, TX, USA), and graphs were produced in GraphPad Prism (v.10, GraphPad Software, Boston, USA).

## RESULTS

### Microdialysis

The mean RR of ganciclovir was 29% after exclusion of outliers (<10% or >50%) and was used to calculate absolute ganciclovir concentrations. Due to occasional catheter malfunction as well as untimely death of one animal, data could not be recovered from 42 out of 738 samples. This mostly affected the ventricular compartment, meaning that the ventricular T_1/2_ could not be calculated for the first dose.

### PK parameters

**Fig 1:**
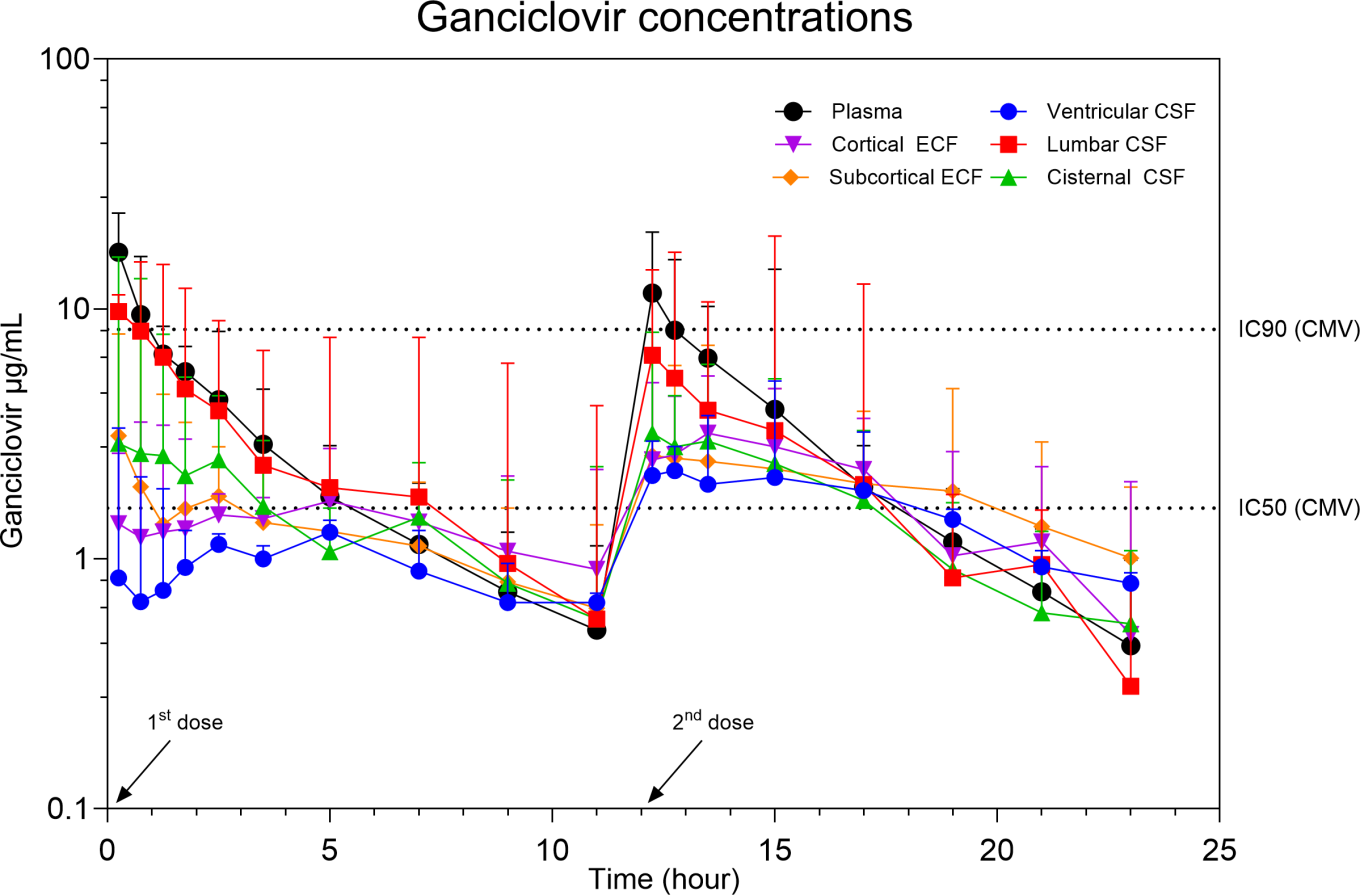
Median concentrations in plasma, ventricular CSF, lumbar CSF, cisternal CSF, cortical ECF, and subcortical ECF in healthy adult pigs after two intravenous bolus infusions of 5 mg/kg ganciclovir at an interval of 12 h. IC_50_ is defined as 1.6 µg/mL and IC_90_ is defined as 8.3 µg/mL.

Concentration time curves of each compartment are presented in Fig. 1. The PK parameters for the first and second dose of each compartment are presented in Table 1. The greatest C_max_ and AUC in the CNS were achieved in the lumbar compartment. No compartment achieved complete tissue penetration. The R_ac_ was >1 in the ventricular and cisternal CSF and both ECF compartments.

**TABLE 1:** PK parameters for the first and second dose of ganciclovir in plasma, ventricular CSF, lumbar CSF, cisternal CSF, cortical ECF, and subcortical ECF^*a*^^,*b*^

	First dose			Second dose		
Parameter	N	Median	Range	N	Median	Range
C_max_ (µg/mL)						
Plasma	6	16.56	9.44–24.25	6	14.78	9.29–20.35
Ventricular	4	1.38	0.67–3.34	5	2.76	2.12–5.15
Lumbar	6	9.93	0.58–15.45	6	6.54	1.13–19.59
Cisternal	6	3.10	1.58–16.14	6	4.03	2.3–8.08
Cortical	6	2.05	1.33–3.53	6	2.37	1.74–5.41
Subcortical	6	3.18	1.3–7.95	6	2.91	1.2–7.16
T_1/2_ (h)						
Plasma	6	2.31	1.97–3.35	6	2.30	2.0–2.84
Ventricular	N/A	N/A	N/A	5	6.11	5.55–8.40
Lumbar	4	3.45	2.27–7.02	6	3.95	2.35–73.09
Cisternal	5	4.12	2.85–6.02	5	3.61	2.98–4.46
Cortical	2	4.17	4.15–4.19	5	6.45	3.33–22.96
Subcortical	6	7.39	3.39–53.15	6	6.74	4.19–11.08
AUC (h × µg/mL)						
Plasma	6	31.13	26.76–40.21	6	30.09	25.72–46.84
Ventricular	4	10.6	5.6–11.03	5	17.18	15.28–27.93
Lumbar	6	28.0	4.80–87.05	6	24.31	8.03–73.05
Cisternal	6	14.16	11.44–33.66	6	16.42	8.99–33.24
Cortical	6	14.42	11.71–18.65	6	17.70	7.85–37.95
Subcortical	6	14.07	8.35–23.2	6	18.65	4.51–43.82
AUC_tissue_/AUC_plasma_						
Ventricular	4	0.35	0.18–0.41	5	0.57	0.5–0.6
Lumbar	6	0.77	0.15–2.87	6	0.57	0.29–2.84
Cisternal	6	0.49	0.35–1.02	6	0.60	0.29–1.02
Cortical	6	0.45	0.35–0.61	6	0.53	0.31–1.37
Subcortical	6	0.42	0.25–0.76	6	0.63	0.18–1.58
AUC_second dose_/AUC_first dose_						
Plasma	5	0.90	0.80–1.75			
Ventricular	4	2.24	1.42–2.95			
Lumbar	5	0.96	0.66–1.67			
Cisternal	5	1.14	0.29–2.36			
Cortical	5	1.27	0.42–2.58			
Subcortical	5	1.62	0.19–3.01			

^*a*^ AUC_0–12_ = area under the concentration-time curve from 0 to 12 h, C_max_ = peak drug concentration, AUC_tissue_/AUC_plasma_ = area under the concentration-time curve ratio of tissue drug/plasma drug. AUC_0–12,12–24_, T_½_, C_max_, and AUC_second dose/first dose_ (R_ac_) are given as medians.

^*b*^ Ganciclovir was administered intravenously as two weight-adjusted doses (5 mg/kg).

#### C_max_

Median CSF compartments ranged from 1.38 to 9.93 (µg/mL) and the ECF compartments from 2.05 to 3.18. Plasma ranged from 9.29 to 24.25. For both the first and second dose, the lumbar C_max_ was higher than in the cortical, subcortical, and ventricular compartments (*P* < 0.01). Furthermore, in the first dose, the ventricular C_max_ was lower than the cisternal, cortical, and subcortical compartments (*P* < 0.01), and the subcortical C_max_ was higher than the cortical ECF and cisternal CSF (*P* < 0.01). In the second dose, the cisternal C_max_ was higher than in the cortical ECF (*P* < 0.01).

#### Area under the curve

Median CSF compartments ranged from 10.6 to 28.0 (h × µg/mL) and the ECF from 14.07 to 18.65. Plasma concentrations ranged from 25.72 to 46.84. In the first dose, the lumbar AUC was higher than the ventricular, cisternal, cortical, and subcortical compartments (*P* < 0.01), and the ventricular AUC was lower than in the cisternal and cortical compartments (*P* < 0.01). The cortical AUC was higher than in the cisternal and subcortical compartments (*P* = 0.02, *P* < 0.01).

In the second dose, the ventricular AUC was lower than in the cortical and subcortical compartments (*P* < 0.01).

#### Penetration ratio

Median CSF compartments ranged from 0.15 to 2.87 and the ECF from 0.18 to 1.58. In the first dose, the lumbar penetration ratio was higher than the ventricular, cisternal, and subcortical compartments (*P* < 0.01), and the cisternal penetration ratio was higher than the ventricular, cortical, and subcortical compartments (*P* ≤ 0.01).

In the second dose, the subcortical penetration ratio was higher than in the cortical and ventricular compartments (*P* < 0.01).

#### First vs second dose

When comparing PK parameters in the same compartment between the first and second dose, the cisternal (*P* = 0.05), cortical, and ventricular compartments (*P* < 0.01) achieved significantly higher C_max_ values in the second dose. The AUC was higher in the ventricular, cortical, and subcortical compartments (*P* < 0.01) in the second dose. The penetration ratio of the ventricular and subcortical compartments was higher in the second dose (*P* < 0.01).

### PK/PD target: t > IC_50_ and IC_90_

The approximate t > IC_50_s are presented in Table 2. Ganciclovir concentrations >IC_50_ were achieved in all compartments. From the first to the second dose, the median t > IC_50_ tended to increase for ventricular CSF (0% vs 50.8%), cisternal CSF (27.5% vs 45.8%), cortical ECF (20.0% vs 50.8%), and subcortical ECF (23.3% vs 55.8%). The t > IC_50_ for lumbar CSF did not increase (64.2% vs 49.2%) in the second dose. No significant differences in t > IC_50_ between the first and second dose were found (*P* > 0.05).

**TABLE 2:** Time > IC_50_ for the first and second dose of ganciclovir in ventricular CSF, lumbar CSF, cisternal CSF, cortical ECF, and subcortical ECF^*a*^

	First dose			Second dose		
Compartment	N	Median	Range	N	Median	Range
Ventricular	4	0	0–1.3	5	6.1	4.5–6.7
Lumbar	6	7.7	0–12	6	5.9	0.8–12
Cisternal	6	3.3	0–12	6	5.5	4–11
Cortical	6	2.4	0–7.9	6	6.1	4.7–12
Subcortical	6	2.8	0–8.1	6	6.7	3.3–12

^*a*^ Values are calculated through interpolation and reported as hours for each 12 h sampling interval.

Concentrations >IC_90_ were only achieved in plasma and lumbar CSF.

## DISCUSSION

To our knowledge, this is the first *in vivo* CNS microdialysis study measuring ganciclovir continuously in CSF, brain ECF, and plasma. The median C_max_ ranged from 1.38–9.93 (µg/mL) in the CSF and 2.05–3.18 in the brain ECF. The median AUCs ranged from 10.6–28.0 (h × µg/mL) in the CSF and 14.07–18.65 in the brain ECF. All compartments, except the lumbar CSF, had greater penetration ratios after the second dose. The C_max_, AUC, and t > IC_50_ showed a trend toward accumulation in the cisternal and ventricular CSF compartments and both ECF compartments after the second dose. Concentrations >IC_90_ were only achieved in plasma and lumbar CSF.

Animal studies of homogenized brain tissue from mice and rats have reported peak ganciclovir concentrations of 0.60–3.96 µg/g (36, 37) as well as an AUC_0–6_ of 0.664 µg × h/mL (36) and AUC_0–t_ of 2.25 µg × h/g^-1^ (37). Ventricular CSF peak concentrations of 0.7 µg/mL and an AUC of 2.8 µg/mL × h have also been reported in rhesus monkeys (32). The results from homogenized brain tissue (36, 37) are reported as µg/g which is not directly comparable to µg/mL obtained from liquids such as CSF or ECF. Furthermore, these studies may carry a risk of overestimating drug concentrations due to the presence of small blood vessels within the tissue.

A human microdialysis study by Peredo et al. measured brain ECF concentrations for 12 h after a single oral dose (900 mg) of valganciclovir. They reported similar PK values to this study, with a C_max_ of 2.6 µg/mL, a T_1/2_ of 4.5 h and an AUC_0-12_ of 15.8 µg × h/mL. The sampling was performed in adjacent brain tissue after resection of a glioblastoma (46), meaning the integrity of the blood-brain-barrier (BBB) probably was severely compromised. It is uncertain whether the BBB during viral encephalitis is disrupted to the same degree. Other human studies have reported C_max_ values of 0.5–5.8 µg/mL, a T_1/2_ of 3.5 h and penetration ratios of 0.135–3.4 in unspecified CSF compartments of patients with normal renal function (33–35). To our knowledge, no studies of ganciclovir in the CNS have been performed during steady state.

The high ganciclovir concentrations in the lumbar CSF might be partially explained by the production and natural flow of CSF from the choroid plexus, through the third and fourth ventricles, and from there to the CSF over the convexities (approx 2/3) and into the spinal canal (approx 1/3) resulting in a reduced CSF flow in the lumbar subarachnoid space thereby increasing drug diffusion into lumbar CSF. Consequently, in humans after IV injection, most drugs achieve higher concentrations in the lumbar than in the ventricular CSF (47). CSF in the porcine spinal cord has recently been found to have a lower pulsatile flow and slower velocity wave propagation compared to humans (48). However, it remains unknown whether and how this influences drug pharmacokinetics in the CSF.

PK studies investigating other drugs with higher molecular weight and varying lipophilicity have reported similar overall CNS drug distribution patterns as this study with higher lumbar concentrations compared to the brain ECF and lateral ventricles (38, 49). This pattern of low concentrations of ganciclovir in brain ECF and ventricular CSF may be problematic, considering that CMV CNS disease in AIDS patients often presents as periventricular encephalitis (1, 50). Previous studies have exclusively utilized single point estimates by lumbar punctures as a proxy for total drug exposure in the CNS (33–35), but our novel observations indicate that lumbar CSF might overestimate the drug exposure in other CNS compartments. These findings are consistent with De Lange et al.’s understanding of pharmacokinetics between CSF and the brain parenchyma, which states that lower drug concentration in the ECF compared to the CSF compartments would be expected since the blood-CSF barrier is slightly more permeable for hydrophilic drugs such as ganciclovir than the BBB (51). That being said, Hugh Davson et al. might argue that “sink action” would result in greater ECF drug concentrations than in the CSF (52) which fits the pattern of lower ventricular drug concentrations than in the cortical and subcortical compartments. Considering our sample size (*n* = 6) and the fact that some data were lost from the ventricular compartment, it is unclear which theory is most likely to be correct. Thus, this phenomenon needs to be investigated in future studies.

Due to the high pH and poor gastro-intestinal absorption of ganciclovir, it is typically administered as an IV infusion over 1 h. Considering that our study administered each dose as a bolus, a lower C_max_ and slower achievement of therapeutic levels would be expected in the clinical setting, compared to our study findings. Conversely, patients suffering from viral encephalitis might have an inflamed BBB, which may be more permeable to hydrophilic drugs, contributing to a greater drug penetration ratio at the site of inflammation (51). This emphasizes the need for more thorough human PK studies in order to better understand the intricacies of brain pharmacokinetics in the clinical setting.

The R_ac_ > 1 as well as a greater AUC_0–12_ and t > IC_50_ in the second dose compared to the first dose all suggest an accumulation of ganciclovir in both the ECF and CSF compartments. In contrast, Fletcher et al. found no apparent drug accumulation in the CSF of two human patients with CMV pneumonitis or retinitis (34). However, their observations were based on a total of four CSF samples taken at varying time points.

Due to the lack of an established PD/PK model for CMV- and HHV-6b encephalitis, it is unclear whether drug levels >IC_50_ or IC_90_ would have the desired clinical efficacy. While the primary target of our study was the IC_50_, it has previously been discussed in the literature whether the IC_90_ might be more clinically relevant since it better reflects what is aimed for during treatment (24). Assuming that concentrations >IC_90_ are therapeutic, our data suggest that an increase in the first dose of ganciclovir may allow for more rapid obtainment of adequate CNS concentrations. Considering that established risk factors of ganciclovir resistance include insufficient therapeutic drug response and prolonged drug therapy (53, 54), a rapid obtainment of greater drug concentrations may help reduce the risk of ganciclovir resistance and could potentially improve the clinical outcome. That being said, there is currently no evidence that reaching the IC_90_ rather than the IC_50_ would result in a significantly higher viral inhibition, and whether the potential benefit would outweigh the risk of severe side effects. PD/PK modelling of our current data could assist clinicians in determining the relationship between tissue drug levels and antiviral effect, which may help with future dosage optimization.

When modifying ganciclovir treatment, the clinician should be cautious of myelosuppression and neurotoxicity. Myelosuppression seems to be mostly associated with long-term treatment (55–57), meaning that a greater induction dose might only cause a negligible increase in myelosuppression. Janoly-Dumenil et al. presented a predictive pharmacodynamic model, based on *in vitro* ganciclovir plaque assays, which suggests that IV infusions twice the current recommended dose (10 mg/kg every 12 h) for the first 2 days would be ideal for maximizing antiviral effect, without causing notable suppression of lymphoblastoid cells (55). Finally, incidents of neurotoxicity have been reported in ganciclovir- and acyclovir-treated patients suffering from renal dysfunction (35, 58, 59), meaning the clinician should be cautious with dosing in these patients.

### Strengths and limitations

This animal model performs simultaneous continuous drug measurements in both ECF, CSF, and plasma as opposed to point sampling, thus providing an extensive picture of the total drug exposure in the CNS. ECF measurements of antiviral drugs are considered a preferable target compared to CSF due to ECF being closer to the antiviral drugs' intracellular site of action.

The upper limit of the CMV IC_50_ was utilized, which runs the risk of underestimating the therapeutic effects of ganciclovir in the CNS. A dose of 5 mg/kg was chosen to best emulate the current dosing regimen, but allometric scaling is typically utilized when converting doses between animals and humans. The converting factor of pigs the size in our current study would be 1.1 (5.5 mg/mL) (60), suggesting that our study might slightly underestimate equivalent human doses.

The porcine brain is structurally and functionally close to the human brain compared to other vertebrates, regarding cortical organization, anatomical structures, and gray- and white-matter ratio (61). However, there are obvious differences between humans and pigs that may affect the generalizability of the results to human conditions. The porcine spinal cord is, for example, horizontally positioned, which may have unknown effects on cardiovascular circulation as well as CSF flow and turnover (38). Furthermore, a non-inflamed porcine brain does not reflect the pathophysiological conditions of human patients with acute encephalitis, with BBB impairment and potential effects on influx- and efflux-pump activity (62, 63). To simulate these pathological conditions, other studies have applied methods of inducing inflammation in the CNS of pigs via inoculation of pro-inflammatory molecules into the CSF with promising results (64–67), that may be incorporated into future PK brain studies. A physiologically based pharmacokinetic (PBPK) model (68) could be utilized to account for interspecies differences and the inflammatory conditions during acute encephalitis. Furthermore, simultaneous human brain ECF concentrations could potentially be predicted from the single CSF measurements found in the current literature. To our knowledge, no PBPK model has yet been established for the porcine brain, but it would be a valuable addition to our animal PK data in the future.

Finally, due to the correction of RR as well as equipment malfunction and analytical errors, some data were lost from this study. Specifically, data from the ventricular compartment is lacking due to catheter malfunction and the death of one animal before completion of the sampling procedure.

### Conclusion

We present a porcine model comparing the CNS concentrations and distribution of ganciclovir in the CSF and ECF, respectively. Our results suggest a heterogeneous distribution between each CSF and ECF compartment. This study found a cumulative pattern in the ECF compartments, suggesting that long-term treatment might achieve therapeutic levels. We propose that a higher loading dose might be beneficial to achieve therapeutic levels faster, hopefully improving the clinical outcomes in patients suffering from CMV- and HHV-6b encephalitis. These considerations need to be examined in future studies.

## AFTER EPUB

*[This article was published on 21 March 2025 with errors in the abstract and affiliations. The abstract was corrected on 28 March 2025, and the affiliations were corrected in the current version, posted on 3 April 2025.]*

## SUPPLEMENTAL MATERIAL

Supplemental material aac.01815-24-s0001.docx Description of HPLC analysis and overview of microdialysis/plasma sampling intervals.

ASM does not own the copyrights to Supplemental Material that may be linked to, or accessed through, an article. The authors have granted ASM a non-exclusive, world-wide license to publish the Supplemental Material files. Please contact the corresponding author directly for reuse.
