# Supplementary material for: Distribution of ganciclovir in the porcine central nervous system
Source: Antimicrob Agents Chemother. 2025 Mar 21;69(5):e01815-24. doi: 10.1128/aac.01815-24 (PMC12057358; doi:10.1128/aac.01815-24)
Supplement: Supplemental material — Description of HPLC analysis and overview of microdialysis/plasma sampling intervals. [file aac.01815-24-s0001.docx]

# Supplementary Material

**Quantification of ganciclovir concentrations**

Standards and quality controls (QCs) for ganciclovir dialysate samples were made through dilution of stock solution in NaCl (0.1%). For the plasma samples blank porcine plasma was diluted in NaCl. Calibration standards were determined at concentrations of 10, 50, 125, 250, 500 and 1000 µg/mL. QCs were prepared at concentrations of 60 (or 80), 450 and 800 µg/mL in both matrices.

**Sample preparation**

*Dialysate samples*

All dialysate samples, including QCs and calibration standards, were thawed at room temperature prior to analysis. Blanks (dialysate matrix without ganciclovir) and double blanks (only water) were also prepared. A solution of the internal standard (benzylpenicillin) and 495 µL of NaCl (0.1%) was added to all each well assigned to standards, QCs and blanks. The double blanks received 495 µL of NaCl (0.1%) without the internal standard. 5 µL of each standard solution and QC was added to their allotted wells, and 5 µL of pure NaCl (0.1%) was added to the blank and double blank wells. Proper mixing was ensured by vortexing all samples for 10 seconds, followed by centrifugation at 500 x g for 5 minutes, to separate any particulates. Afterwards, 300 µL of supernatant from all wells were transferred to a new deep well for plate for LC-MS/MS analysis.

*Plasma samples*

All plasma samples, including QCs and calibration standards were thawed at room temperature prior to analysis. Blank (plasma without ganciclovir) and double blanks (only water) were prepared like the dialysate samples. The internal standard (benzylpenicillin) and 495 µL of NaCl (0.1%) was added to all each well. 5 µL of each standard solution and QC was added to their respective wells, and the blank well received pure plasma, while the double blank wells received 5 µL of pure water.

250 µL of all plasma samples were transferred to their allotted wells, for protein filtering and precipitation. 250 µL of standard and blank solutions were also added to their corresponding wells in the filter plate. To facilitate filtration, the filter place was placed on top of a deep well plate and centrifuged at 200 x g for 10 minutes – centrifugation times were extended accordingly, if the plasma did not sufficiently pass through the filter. 20 µL of filtrate was transferred from each well to a deep well plate, while 200 µL from standards, QCs and blanks were transferred to their corresponding wells in the deep plate. Then 180 µL of internal standard diluted in water was added to all wells containing samples. To ensure homogeneity the plate was lightly shaken for 10 seconds and then centrifuged to eliminate residual air bubbles prior to LC-MS/MS analysis.

**Liquid Chromatography – Mass spectrometry analysis**

The quantitative analysis of ganciclovir was performed using high-pressure liquid chromatography, coupled with tandem mass spectrometry (HPLC-MS/MS). The system consisted of a Shimadzu Exion UHPLC couple to a SCIEX 4500 QTrap mass spectrometer with an electrospray ionization source. To achieve chromatographic separation a Phenomenex Luna Omega C18 column with a particle size of 1.6 µm, dimensions of 50 x 2.1 mm, and a pore size of 100 Å, maintained at a temperature of 65 °C. In the mobile phase Milli-Q water with 0.1% formic acid (solvent A) and acetonitrile with 0.1% formic acid (solvent B), delivered at a flow rate of 0.6 mL/min.

The gradient elution program was initiated with 10% solvent B at 0.0 minutes. Solvent B proportions were increased linearly to 80% at 0.90 minutes and then increased to 95% at 0.95 minutes and held at 95% to 1.35 minutes. At 1.35 minutes solvent B was then increased to 100%. At 1.35 minutes the composition was returned to the initial 10% solvent B, which was maintained until 1.60 minutes (end of runtime per sample). The injection volume of was 10 µL for all samples. Mass spectrometric detection was performed in positive electrospray ionization mode using multiple reaction monitoring (MRM). The source parameters were optimized with a curtain gas setting of 40 psi, an ion spray voltage of 5,500 V, a temperature of 450 °C, and both ion source gas 1 and ion source gas 2 set at 30 psi. The entrance potential was set to 10 V.

**Method validation**

Validation of the analytical model was performed in regard to sensitivity, linearity, accuracy and precision. The calibration curves demonstrated linearity over the range of 10-1000 µg/mL with correlation coefficients (R^2^) exceeding 0.99. Inter- and intra-day variability were determined by analyzing the QC samples at 60 (or 80), 450 and 800 µg/mL in triplicates over multiple days. Coefficients of variation (CV%) were found to be less than 5% across all QC levels, suggesting high accuracy and reproducibility. The LLOQ for ganciclovir was determined to be 0,01 µg/mL.

**Overview of sampling intervals after the 1^st^ and 2^nd^ dose:**

| **Microdialysis sampling** | **Plasma sampling** |
| --- | --- |
| **Minutes post dose** | **Minutes post dose** |
| 1^st^ dose | 1^st^ dose |
| 30 | 15 |
| 60 | 45 |
| 90 | 75 |
| 120 | 105 |
| 180 | 150 |
| 240 | 210 |
| 360 | 300 |
| 480 | 420 |
| 600 | 540 |
| 720 | 660 |
| 2^nd^ dose | 2^nd^ dose |
| 30 | 15 |
| 60 | 45 |
| 120 | 105 |
| 240 | 180 |
| 360 | 300 |
| 480 | 420 |
| 600 | 540 |
| 720 | 660 |
